# Delayed inflammatory reaction to hyaluronic acid lip filler after the Pfizer-BioNTech COVID-19 vaccine: A case report

**DOI:** 10.1016/j.heliyon.2023.e18274

**Published:** 2023-07-17

**Authors:** Thikryat Neamatallah

**Affiliations:** Department of Pharmacology and Toxicology, Faculty of Pharmacy, King Abdulaziz University, Jeddah 21589, Saudi Arabia

**Keywords:** COVID-19, Delayed hypersensitivity, Hyaluronic acid, Dermal filler, Pfizer-BioNTech

## Abstract

Hypersensitivity reactions can be a side effect to any vaccine, but they are usually rare. The COVID-19 vaccination may cause hypersensitivity, and several cases of delayed hypersensitivity (DH) to hyaluronic acid (HA) dermal filler have been documented. The current report presents a case of a 36-year-old female patient with DH to HA dermal filler after receiving the Pfizer-BioNTech COVID-19 vaccine. Symptoms, including dryness, swelling, and a painless nodule, appeared after the first and second doses of the vaccine. The patient was treated with intralesional hyaluronidase and triamcinolone in the outpatient clinic. Although HA is relatively safe and routinely used in aesthetic medicine, DH reactions must be considered. Therefore, an appropriate patient history should be obtained, and physicians should provide counselling on the potential reactions to avoid these adverse effects.

## 1 Introduction

Nonsurgical aesthetic treatments using hyaluronic acid (HA) for soft tissue fullness augmentation, such as lip augmentation, have become popular. Considering the safety and effectiveness, different HA products are currently the most often applied soft-tissue fillers [1,2]. Complications from HA filler are rare; however, if presented, they are classified according to the timing of appearance: early (within 14 days), late (>14 days to 1 year), and delayed (after 1 year) [3,4]. Acute hypersensitivity reactions are immunoglobulin E (IgE)-mediated, whereas delayed hypersensitivity (DH) reactions exist as swelling, erythema, and induration are mediated by T lymphocytes rather than antibodies [1,4]. Possible triggers to HA-delayed reactions might be exposure to an infection or trauma, as well as injection-related factors, such as repeated therapy, filler volume, properties, or components [4,5].

The delayed HA hypersensitivity reaction has been observed recently with individuals infected with SARS-CoV-2, which causes Coronavirus Disease 2019 (COVID-19), or immunized with COVID-19 vaccines [6,7]. Herein, we describe a case of late-onset inflammatory reaction to HA lip filler after exposure to the first dose of the mRNA Pfizer-BioNTech COVID-19 vaccine.

## 2 Case report

**Fig. 1:**
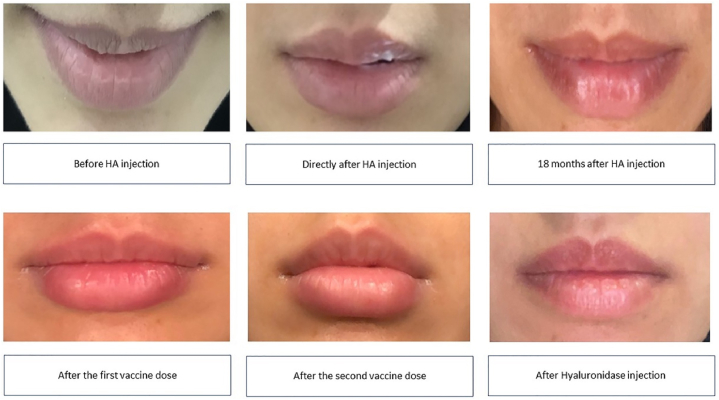
Images taken by the patient's iPhone camera showing delayed hypersensitivity reactions to HA dermal filler after the COVID-19 vaccination.

A 36-year-old healthy Saudi female weighing 49 kg (BMI = 20.7 kg/m2) was injected with 1 mL of Juvéderm Volfit (Allergan Inc.) HA soft filler to her lips at a private clinic (in Jeddah, Saudi Arabia) in May 2019. The patient had clear medical history and allergies to medications. She did not suffer from any complications afterward and lips filler lasted for 6 months. In March 2021, the patient received her first dose of the mRNA Pfizer-BioNTech COVID-19 vaccine. Three days later, she reported dryness, induration, and erythematous swelling in the lower lip. Fig. 1 presents a selfie image of the reported observations and symptoms taken by the patient (The patient signed on a consent form for the use of her images and all the required data for publication). In the following days, the patient tried to moisturize her lips and avoided using cosmetic products. The patient did not use any medications and the symptoms reduced within two weeks. The second dose of the mRNA Pfizer-BioNTech COVID-19 vaccine was administered to the patient three weeks after the first one. The following day (within 12 hours) the patient complained of the same symptoms and continued at intermittent periods, specifically in the morning. Swelling and dryness were getting worse by day 7, thus the patient visited her doctor on day 10 (May 2021), who injected 15 units of hyaluronidase and 7 mg of triamcinolone directly into the lesion. Consequently, the patient experienced an improvement in the following days with no further symptoms.

## 3 Discussion

Several reports of DH reactions to facial fillers have been documented following vaccination with mRNA COVID-19 vaccines [[7], [8], [9]]. These reactions are generally characterised by localized redness and swelling. They can be reported in cases where HA filler was injected weeks or even months prior to vaccination [10]. *M*-RNA COVID-19 vaccines are synthetic molecules of the RNA sequence that encodes the viral spike (S) glycoprotein of SARS-CoV-2 [11]. The reaction to these vaccines is mostly T-cell-mediated, in which the cells are recruited into the tissue to produce cytokines that initiate local inflammation. It is suggested that fillers may act as adjuvants rather than direct T-cell activators, enhancing the antigen-specific immune response [10,12,13]. Hyaluronic acid (HA) used in dermal fillers starts degrading after 3–5 months of injection to produce breakdown products that expose the immune system to unknown antigens, which can be triggered by various factors such as vaccines. This can potentially cause an immune response and DH reactions [10,14]. Symptoms of DH can be self-limiting within days to weeks; however, it might require medical intervention for the appropriate resolution of the symptoms [7,8]. The use of antihistamines was found of limited benefits [4,15]. However, a single intravenous dose of diphenhydramine has been used in some cases to control DH symptoms [5]. Injecting steroids combined with hyaluronidase into the inflamed lesions is considered the first-line approach [15,16]. Hyaluronidase acts to dissolve visible nodules of HA [3,17]. In severe cases, the patient should be admitted to the emergency department, where analgesics are provided to control the pain. In addition, a short-course of oral steroid is highly recommended [3,4,17]. For example, prednisone is commonly prescribed between 30 and 60 mg daily, with dose reduction over 5 days, depending on symptom severity [3,17,18]. One study suggested the use of oral angiotensin-converting enzyme (ACE) inhibitor e.g. lisinopril 5–10 mg/. The proposed mechanism is that the ACE inhibitor would reduce the angiotensin II‐induced pro‐inflammatory pathway [11].

In the current case report, the symptoms of a delayed type of hypersensitivity reaction to HA developed three days after the first vaccine dose and within 12 hours after the second one. According to Naranjo scale the causality assessment method for assessing drug induced adverse events, DH reaction to the injected filler was most probable (total score 7) (Appendix 1) [19]. Other possible causative factors like trauma, infection or prior allergies were excluded. Thus, it is suggested that COVID-19 vaccine triggered a pro-inflammatory response at the site of HA filler. According to the timeline of receiving the filler and both vaccine doses, DH symptoms appeared during the peak antibody response to the Pfizer vaccine (week to 20 days). The symptoms resolved spontaneously after the injection of hyaluronidase and triamcinolone. Previous cases of facial swelling were managed similarly using hyaluronidase and steroids [5,20,21]. Kalantari et al. mentioned nine reports of swelling at the site of cosmetic fillers after Moderna and Pfizer COVID-19 vaccination [7]. Out of these reports, a single case showed a lip filler reaction after getting the second dose of the Pfizer vaccine. The interval between filler injection and second dose vaccine injection was 13 months. The patient was treated with methylprednisolone and reductions in the swelling and the pain was noticeable after 5 days [20]. The benefits of the COVID-19 vaccination are of greater value, compared to the risk of dermal DH reactions. Therefore, it is suggested that patients wait 3 weeks after completing the vaccine series before undergoing any elective cosmetic procedures, including lip filler injections. The peak antibody response to mRNA vaccines typically occurs approximately two to three weeks after the second vaccine dose or around five to six weeks after the vaccination process begins [22]. Additionally, patients must be appropriately counselled on the potential side effects. Physicians need to clarify the limitations, and the possible complications of dermal fillers, thus patients' expectations and side effects can be managed. In addition, the contraindications and the suitability of different fillers need to be discussed as filler's constituents or excipients might cause severe allergies to those with a history of anaphylaxis. An appropriate consent form prior to filler use should be signed by the patient. This is to avoid dissatisfaction and to ensure following the physician's recommendations after the treatment [23,24].

## Author contribution statement

All authors listed have significantly contributed to the investigation, development and writing of this article.

## Data availability statement

Data included in article/supplementary material/referenced in article.

## Declaration of competing interest

The authors declare that they have no known competing financial interests or personal relationships that could have appeared to influence the work reported in this paper.
