# Supplementary material for: Delayed inflammatory reaction to hyaluronic acid lip filler after the Pfizer-BioNTech COVID-19 vaccine: A case report
Source: Heliyon. 2023 Jul 17;9(7):e18274. doi: 10.1016/j.heliyon.2023.e18274 (PMC10395472; doi:10.1016/j.heliyon.2023.e18274)
Supplement: Multimedia component 1 [file mmc1.docx]

| **Naranjo Adverse Drug Reaction Probability Scale** | | | | |
| --- | --- | --- | --- | --- |
| **Question** | Yes | No | Do Not  Know | Score |
| 1. Are there previous *conclusive* reports on this reaction? | +1 | 0 | 0 |  |
| 2. Did the adverse event appear after the suspected drug was administered? | +2 | ‐1 | 0 |  |
| 3. Did the adverse reaction improve when the drug was discontinued or a  *specific* antagonist was administered? | +1 | 0 | 0 |  |
| 4. Did the adverse event reappear when the drug was re‐administered? | +2 | ‐1 | 0 |  |
| 5. Are there alternative causes (other than the drug) that could on their own have caused the reaction? | ‐1 | +2 | 0 |  |
| 6. Did the reaction reappear when a placebo was given? | ‐1 | +1 | 0 |  |
| 7. Was the drug detected in blood (or other fluids) in concentrations known to be toxic? | +1 | 0 | 0 |  |
| 8. Was the reaction more severe when the dose was increased or less severe when the dose was decreased? | +1 | 0 | 0 |  |
| 9. Did the patient have a similar reaction to the same or similar drugs in *any*  previous exposure? | +1 | 0 | 0 |  |
| 10. Was the adverse event confirmed by any objective evidence? | +1 | 0 | 0 |  |
| **TOTAL SCORE:** | | | |  |


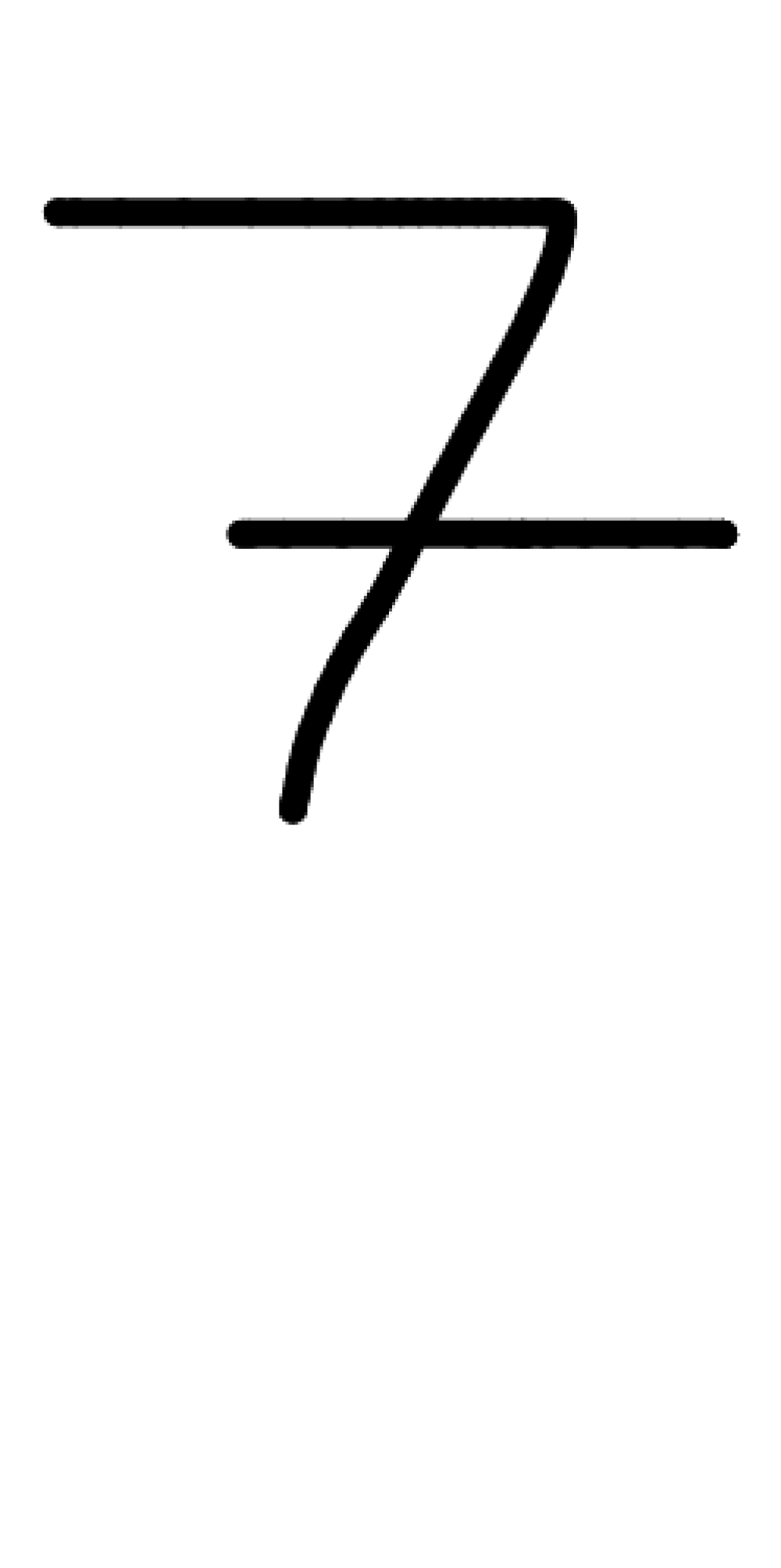
Modified from: Naranjo CA et al. A method for estimating the probability of adverse drug reactions. Clin Pharmacol Ther 1981; 30: 239­245.

**Naranjo Algorithm - ADR Probability Scale**

| **Score** | **Interpretation of Scores** |
| --- | --- |
| **Total Score**  **≥9** | **Definite**. The reaction (1) followed a reasonable temporal sequence after a drug or in which a toxic drug level had been established in body fluids or tissues, (2) followed a recognized response to the suspected drug, and (3) was confirmed by improvement on withdrawing the drug and reappeared on reexposure. |
| **Total Score 5 to 8** | **Probable**. The reaction (1) followed a reasonable temporal sequence after a drug, (2) followed a recognized response to the suspected drug, (3) was confirmed by withdrawal but not by exposure to the drug, and (4) could not be reasonably explained by the known characteristics of the patient’s clinical state. |
| **Total Score 1 to 4** | **Possible**. The reaction (1) followed a temporal sequence after a drug, (2) possibly followed a recognized pattern to the suspected drug, and (3) could be explained by characteristics of the patient’s disease. |
| **Total Score**  **≤0** | **Doubtful**. The reaction was likely related to factors other than a drug. |
